# Decoherence of black hole superpositions by Hawking radiation

**DOI:** 10.1038/s41467-019-08426-4

**Published:** 2019-03-04

**Authors:** Andrew Arrasmith, Andreas Albrecht, Wojciech H. Zurek

**Affiliations:** 1 Theory Division, T-4, MS B213, LANL, Los Alamos, NM 87545 USA; 2 Department of Physics, University of California Davis, Davis, CA 95616 USA; 3 Center for Quantum Mathematics and Physics, University of California Davis, Davis, CA 95616 USA

## Abstract

An environment interacting with a system acquires information about it, e.g. about its location. The resulting decoherence is thought to be responsible for the emergence of the classical realm of our Universe out of the quantum substrate. However, this view of the emergence of the classical is sometimes dismissed as a consequence of insufficient isolation and, hence, as non-fundamental. In contrast to many other systems, a black hole can never be isolated from its Hawking radiation which carries information about its location, making this lack of isolation fundamental. Here we consider the decoherence of a “black hole Schrödinger cat”—a non-local superposition of a Schwarzschild black hole in two distinct locations—due to its Hawking radiation. The resulting decoherence rate turns out to be given by a surprisingly simple equation. Moreover, and in contrast to known cases of decoherence, this rate does not involve Planck’s constant *ħ*.

## Abstract (web summary)

A black hole’s Hawking radiation inevitably deposits records of its location in the environment, making isolation fundamentally impossible. Here, the authors investigate the decoherence of a black hole in a superposition of two positions due to its Hawking radiation and give the rate of this process.

## Introduction

Despite nearly a century of effort, the unification of quantum mechanics with general relativity is still a work in progress. Perhaps the most promising breakthrough in investigating the relation between quantum theory and general relativity came when Hawking^1^ (following heuristic arguments of Bekenstein^2^) used quantum field theory to show that Schwarzschild black holes radiate as if they were at a temperature *T*_H_ given by:

1 $$k_{\mathrm{B}}T_{\mathrm{H}} = \frac{{\hbar c^3}}{{8\pi GM}} = \frac{{\hbar c}}{{4\pi R_{\mathrm{S}}}},$$

where *M* is the mass of the black hole and *R*_S_ is the Schwarzschild radius.

Hawking radiation defies the classical expectation that nothing can be emitted from a black hole. It was initially hoped that this result would pave the way to quantization of gravity. However, Hawking radiation has instead deepened the mystery by implicating entropy (and, hence, information) in questions involving quantum theory and gravity (e.g., the black hole information paradox).

The origin of classicality in other settings has been, in the meantime, clarified by the theory of decoherence^3–5^. As in the black hole information paradox, information plays a key role: Decoherence is caused by the information flowing from the system into its environment and the resulting formation of records of its selected observables in that environment^6–8^. It is now widely (though not universally) accepted that the effectively classical behavior of macroscopic systems in our quantum Universe is a consequence of decoherence. Even weak interactions can result in such leakage of information and, consequently, in decoherence. However, information has been traditionally viewed as inconsequential in classical Newtonian physics, and isolation as an experimental difficulty that should be inconsequential for foundations. Therefore, the decoherence-based view of the emergence of the classical in our quantum Universe has been regarded by some^9^ as not fundamental.

Unlike other cases that have been investigated, a black hole cannot be isolated: it creates its own environment—Hawking radiation. Therefore, its decoherence is not just a practical matter, but fundamental. The decoherence of black holes by Hawking radiation thus provides a glimpse into a place where quantum mechanics and general relativity meet in course of a quantum-to-classical transition. Below we obtain and discuss the rate of decoherence of a Schwarzschild black hole in both thermal equilibrium with a radiation bath as well as in a vacuum and discuss the implications of our results.

## Results

### Decoherence in thermal equilibrium

**Fig. 1:**
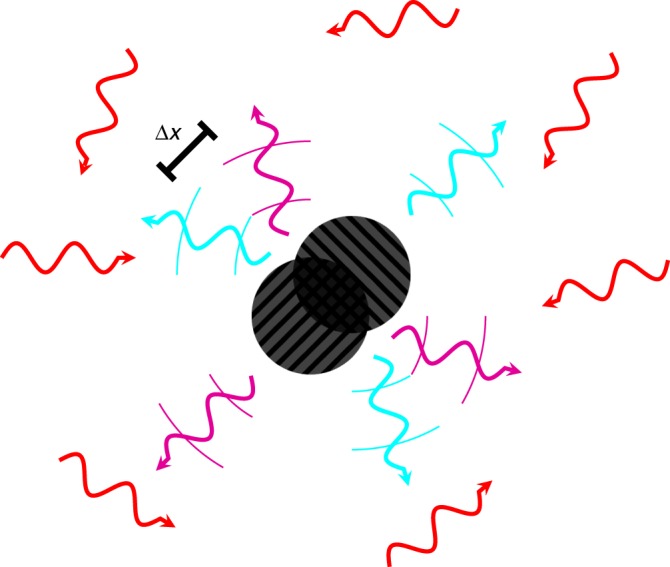
A black hole in a superposition of two position states with separation Δ*x*, immersed in a thermal bath of massless quanta. The quanta from the thermal bath are shown in red. Information about the location of the black hole is carried off by scattered and emitted quanta, shown in cyan and purple for the two different positions

We first consider the case of a Schwarzschild black hole in thermal equilibrium with a radiation bath. Decoherence is caused both by the quanta emitted by the black hole and by the quanta in the external heat bath that are scattered by it (see Fig. 1). The cross section for emission and absorption of Schwarzschild black hole approaches $27\pi R_{\mathrm{S}}^2$ for high energy quanta of massless species (i.e., the geometrical optics limit)^10^. When the wavelength of these quanta becomes comparable to the size of the hole, their behavior becomes more complicated and species-dependent. This is because quanta have to penetrate the potential barrier at ~3*R*_S_ which gives rise to the so-called graybody factors for the modes. For the sake of simplicity, we choose to work in the geometrical optics approximation.

In thermal equilibrium, in each mode, the black hole will radiate back as much as falls into the horizon, making the information exchange from absorption and emission essentially the same as a scattering from a dielectric sphere from the point of view of decoherence. We therefore adapt the decoherence formalism for photon scattering from dielectric spheres to our current case. The decoherence rate of a dielectric sphere that starts in a superposition of two locations separated by Δ*x* and is immersed in a radiation heat bath at temperature *T* in the dipole approximation (${\mathrm{\Delta }}x \ll \lambda$, where *λ* is the dominant wavelength of the radiation) is given by^5,11,12^:

2 $$\tau _{\mathrm{D}}^{ - 1}({\mathrm{\Delta }}x) = \left( {16\frac{{8!\zeta (9)}}{{9\pi }}} \right)\frac{{\tilde a^6{\mathrm{\Delta }}x^2(k_{\mathrm{B}}T)^9}}{{c^8\hbar ^9}}.$$

Above $\tilde a$ is the effective radius of the sphere. Adapting this for a Schwarzschild black hole in a radiation bath at temperature *T* = *T*_H_, we set $\tilde a^2 \simeq 27R_{\mathrm{S}}^2$. The resulting decoherence rate is then:

3 $$\begin{array}{*{20}{c}} {\tau _{\mathrm{D}}^{ - 1}({\mathrm{\Delta }}x)} \hfill & = \hfill & {\left( {16\frac{{8!\zeta (9)}}{{9\pi }}} \right)\frac{{27^3}}{{(4\pi )^9}}\left( {\frac{{{\mathrm{\Delta }}x}}{{R_{\mathrm{S}}}}} \right)^2\left( {\frac{c}{{R_{\mathrm{S}}}}} \right)} \hfill \\ {} \hfill & = \hfill & {d\left( {\frac{{{\mathrm{\Delta }}x}}{{R_{\mathrm{S}}}}} \right)^2\left( {\frac{c}{{R_{\mathrm{S}}}}} \right),} \hfill \end{array}$$

where *d* ≃ 0.0576. This is a surprisingly simple expression and is expected to a be good approximation when |Δ*x*| < *R*_S_. The total rate will be proportional to the number of such species, and will have to be suitably modified for massive quanta.

Neither the above decoherence rate nor the corresponding decoherence time:

4 $$\tau _{\mathrm{D}}({\mathrm{\Delta }}x) = d^{ - 1}\left( {\frac{{R_{\mathrm{S}}}}{{{\mathrm{\Delta }}x}}} \right)^2\left( {\frac{{R_{\mathrm{S}}}}{c}} \right) \simeq 17.37\left( {\frac{{R_{\mathrm{S}}}}{{{\mathrm{\Delta }}x}}} \right)^2\left( {\frac{{R_{\mathrm{S}}}}{c}} \right),$$

depend on the Planck constant *ħ*. This is unusual as other decoherence rates and times generally depend on *ħ*. Here, however, the decoherence timescale in the natural black hole units [*R*_S_/*c*] is simply the square of the distance in natural units [*R*_S_]. Planck’s constant, quantum theory’s defining constant, unexpectedly disappears. Since the basic timescale here is set by the “light-crossing time” *R*_S_/*c*, superpositions of larger black holes would decohere more slowly when separated by the same distance, or even by the same fraction of their Schwarzschild radius. This may seem surprising (usually larger systems decohere faster), but the temperature of the radiation responsible for decoherence decreases with black hole size, and this effect dominates.

Two extreme cases (that mark the two likely limits of the range of applicability of Eqs. (3) and (4)) are worth noting. For superpositions Δ*x* > *R*_S_, the dipole approximation (which assumes that the dominant wavelength responsible for decoherence is larger than Δ*x*) breaks down, and the decoherence rate saturates^12^ (i.e., it does not increase with larger separations, as Eqs. (2) and (3) would suggest).

The other interesting case where Eqs. (2) and (4) will likely break down is when Δ*x* becomes equal to Planck length $\ell _p$. In that case the decoherence time is given by:

5 $$\tau _{\mathrm{D}}(\ell _p) = d^{ - 1}\left( {\frac{{R_{\mathrm{S}}}}{{\ell _p}}} \right)^2\left( {\frac{{R_{\mathrm{S}}}}{c}} \right) = \frac{8}{d}\frac{{G^2M^3}}{{\hbar c^4}} \simeq 139\frac{{G^2M^3}}{{\hbar c^4}}.$$

Thus—assuming the black hole stood still^13^—it would be localized to Planck length on a timescale comparable to but somewhat shorter than its lifetime due to evaporation into vacuum:

6 $$t_{{\mathrm{BH}}} = 5120\pi \frac{{G^2M^3}}{{\hbar c^4}}.$$

Both expressions assume a single massless mode, and would change accordingly otherwise.

### Decoherence in a vacuum

Next, let us consider decoherence in the case of emission into a vacuum. Aside from changing from effective scattering to emission, this case differs because the black hole will be evaporating and thus have a time-dependent temperature. However, assuming the black hole is sufficiently large, we can follow the standard quasi-static formalism^1,10^ and say that the black hole will evaporate slowly compared to the decoherence rate. We will also once again use the geometrical optics limit to simplify our calculation.

Under these approximations, for the emission of a single massless species we get a decoherence rate of (see the Methods for details):

7 $$\begin{array}{*{20}{l}} {\tau _{\mathrm{D}}^{ - 1}({\mathrm{\Delta }}x)} \hfill & = \hfill & {\frac{{27c\zeta (3)}}{{8\pi ^4R_{\mathrm{S}}}}} \hfill \\ {} \hfill & {} \hfill & { + \frac{{27ic\left( {\psi ^{(1)}\left( {1 - \frac{{i{\mathrm{\Delta }}x}}{{4\pi R_{\mathrm{S}}}}} \right) - \psi ^{(1)}\left( {1 + \frac{{i{\mathrm{\Delta }}x}}{{4\pi R_{\mathrm{S}}}}} \right)} \right)}}{{8\pi ^3{\mathrm{\Delta }}x}}.} \hfill \end{array}$$

**Fig. 2:**
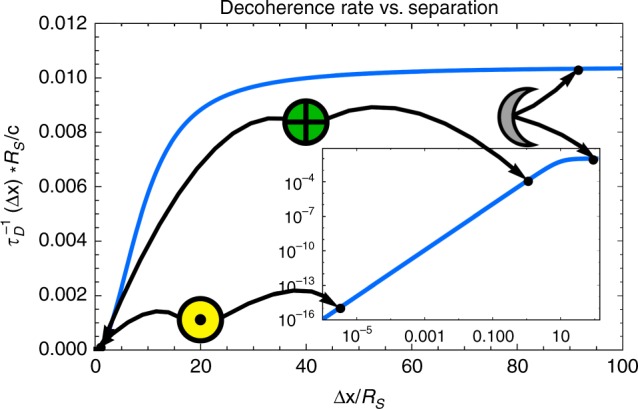
The decoherence rate as a function of Δ*x*/*R*_S_. Note that this levels off for larger separations. Inset is the same function plotted on a log–log scale and showing the decoherence rates that correspond to black hole superpositions with separations of 1 cm if the black hole had the mass of the sun (shown with the yellow sun symbol ⊙), the earth (green earth symbol ⊕), and the moon (gray moon symbol ☾). The mass, Hawking temperature, and decoherence time for these cases are as follows. For the sun *M*_⊙_ = 1.99 × 10^30^ kg, *T*_H⊙_ = 6.17 × 10^−8^ K, and *τ*_D⊙_ = 7.52 × 10^9^ s. For the earth *M*_⊕_ = 5.97 × 10^24^ kg, *T*_H⊕_ = 0.0205 K, and *τ*_D⊕_ = 2.07 × 10^−7^ s. For the moon *M*_☾_ = 7.35 × 10^22^ kg, *T*_H☾_ = 1.67 K, and *τ*_D☾_ = 1.09 × 10^−11^ s

Here *ψ*^(1)^(*y*) is the *n* = 1 polygamma function. (See Fig. 2 for a plot of this function and some test cases.) Note that again, *ħ* does not appear anywhere in this expression.

As with the thermal bath case, we will take a moment here to discuss the limiting cases for this expression. Beginning with the large Δ*x* limit, we can explicitly find the asymptotic saturation discussed in the equilibrium case:

8 $$\mathop {{{\mathrm{lim}}}}\limits_{{\mathrm{\Delta }}x \to \infty } \tau _{\mathrm{D}}^{ - 1}({\mathrm{\Delta }}x) = \frac{{27c\zeta (3)}}{{32\pi ^4R_{\mathrm{S}}}} = {\mathrm{\Lambda }}_{{\mathrm{total}}},$$

where Λ_total_ is the total emission rate for the massless species (see the Methods.) In other words, for sufficiently large separations the decoherence time becomes the time to emit a single quantum.

In the limit that Δ*x* is small compared to *R*_S_, Eq. (7) is approximately:

9 $$\begin{array}{*{20}{l}} {\tau _{\mathrm{D}}^{ - 1}({\mathrm{\Delta }}x \ll R_{\mathrm{S}})} \hfill & \simeq \hfill & {\frac{{27\zeta (5)}}{{256\pi ^6}}\left( {\frac{{{\mathrm{\Delta }}x}}{{R_{\mathrm{S}}}}} \right)^2\left( {\frac{c}{{R_{\mathrm{S}}}}} \right)} \hfill \\ {} \hfill & \simeq \hfill & {1.138 \times 10^{ - 4}\left( {\frac{{{\mathrm{\Delta }}x}}{{R_{\mathrm{S}}}}} \right)^2\left( {\frac{c}{{R_{\mathrm{S}}}}} \right).} \hfill \end{array}$$

We note that, except for a different numerical prefactor, this decoherence rate has the same form as our approximate result for the equilibrium case. Once again, we expect that this result would break down once Δ*x* approaches $\ell _p$, which would give us a decoherence time of:

10 $$\tau _{\mathrm{D}}(\ell _p) = \frac{{8 \ast 256\pi ^6}}{{27\zeta (5)}}\frac{{G^2M^3}}{{\hbar c^4}} \simeq 22400\pi \frac{{G^2M^3}}{{\hbar c^4}}.$$

Note that this is longer than the black hole evaporation time of Eq. (6).

## Discussion

The independence of the decoherence rate on *ħ* is not the result of either the dipole or geometrical optics approximations we used. In particular, the geometrical optics approximation that ignores the details of the graybody factors plays no role for the black hole in the heat bath as Kirchhoff’s Law^14^ mandates that the combination of scattered and emitted quanta induces as much decoherence as if it were indeed a black body emitter. In the case of emission into vacuum, the graybody factors will obviously modify the emitted spectrum, “endowing the black hole with color”. However, these graybody factors depend on the ratio of the wavelength to the Schwarzschild radius in a way that does not introduce any dependence on *ħ*.

This independence of *ħ* contrasts with the “standard lore”^3–5^. For instance, in quantum Brownian motion the decoherence rate is proportional to *γ*(Δ*x*/*λ*_dB_(*T*))^2^, where *γ* is the rate of energy loss and $\lambda _{{\mathrm{d}}{\mathrm{B}}}(T) = \hbar \sqrt {2\pi {\mathrm{/}}(mk_{\mathrm{B}}T)}$ is the thermal de Broglie wavelength. Here *ħ* enters the decoherence rate via its appearance in *λ*_dB_.

Generalizing to black holes with angular momentum and charge (the Kerr–Newman case) also will not introduce any *ħ* dependence for the decoherence rate for massless, uncharged quanta. Aside from breaking spherical symmetry and adjusting the black hole’s radius, this generalization would modify the Bose–Einstein statistics factor for the emissions by adding dependence on the angular mode number *m* and charge e of the emitted quanta: *ω* → *ω* − *m*Ω − eΦ/*ħ*, where Ω is the angular velocity of the event horizon and Φ is the static electric potential from the black hole’s charge evaluated at the event horizon. So long as the particles that are being emitted with these statistics have e = 0, the decoherence rate remains *ħ* independent.

The decoherence rate does, however, depend on *ħ* when the black hole is small enough to emit massive particles. This is because they have a mass-dependent emission probability including a lower frequency cutoff given by $\omega _{{\mathrm{min}}} = \frac{{mc^2}}{\hbar }$. Moreover, the emission of charged particles from a charged black hole has an additional factor of *ħ* in the statistics due to the electromagnetic interaction.

As our paper addresses decoherence of non-local superpositions of black holes, it is natural to inquire whether such superpositions could arise in nature. One conceivable candidate would be three-body systems involving one or more black hole. Such many-body systems can evolve chaotically, so that the initial wavepacket of each body would spread exponentially fast at the rate given by Lyapunov exponents^15^. Binary stars are plentiful, and triplets are also relatively common. Moreover, situations where gravitational interactions give rise to chaotic behavior are known in our solar system^16,17^. Furthermore, LIGO (the Laser Interferometer Gravitational-Wave Observatory) has now detected several black hole mergers^18,19^. As black hole “binaries” seem to be relatively plentiful, it is possible that there may also black hole triplets with chaotic trajectories that would delocalize the individual black holes on the relevant Lyapunov timescale. Thus, while performing a “double slit experiment” with black hole does not seem feasible, dynamics that could lead to non-local superpositions may well be present in astrophysical settings. Moreover, three-body systems with black holes would exert tidal forces, which may stir up internal degrees of freedom of black holes and accelerate decoherence beyond our estimates.

We emphasize that in astrophysical settings decoherence due to environments other than Hawking radiation is likely to overwhelmingly dominate. Thus, even if we ignore accretion disks, magnetic fields, interstellar gas, and other likely environments of astrophysical black holes, the cosmic microwave background (CMB) alone would result in decoherence rate that is ~(*T*_CMB_/*T*_H_)^9^ times faster— ~10^68^ for a solar mass black hole—than what is predicted by Eqs. (3) and (7). This estimate follows directly from Eq. (2). Thus, the decoherence due to Hawking radiation is of interest not because of its ‘practical’ consequences but because of its fundamental nature. That is, for all but the hottest, smallest black holes decoherence in astrophysical settings would be dominated by other environments.

The resulting CMB decoherence rate does depend explicitly on Planck constant. This re-appearance of *ħ* is no surprise: It had disappeared from our earlier expression only due to cancellations that are a direct consequence of the *ħ* dependence of the Hawking temperature, *T*_H_ ~ *ħ*/*M*, Eq. (1).

We close by noting that the lack of dependence of the localization rate—the decoherence rate of spatial superpositions—on *ħ* as well as the remarkable simplicity of the resulting equation may be a consequence of the fact that a black hole is not just an object in space, but is a curved space. One is therefore tempted to speculate that these results hint at (or, perhaps, confirm) an unusual relationship between quantum mechanics and gravity.

## Methods

### Decoherence by radiation

Consider the decoherence of spatial superpositions by radiation emitted into a state |*χ*〉. With the assumption that the state of the emitter is approximately constant, the off diagonal elements of the reduced density matrix of the emitter’s position state get suppressed as:

11 $$\rho ({\boldsymbol{x}},{\boldsymbol{x}}\prime )\prime = \rho ({\boldsymbol{x}},{\boldsymbol{x}}\prime )\left\langle {\chi ({\boldsymbol{x}}\prime )|\chi ({\boldsymbol{x}})} \right\rangle .$$

The change in the reduced density matrix is then:

12 $$\rho ({\boldsymbol{x}},{\boldsymbol{x}}\prime )\prime - \rho ({\boldsymbol{x}},{\boldsymbol{x}}\prime ) = - \rho ({\boldsymbol{x}},{\boldsymbol{x}}\prime )\left( {1 - \left\langle {\chi ({\boldsymbol{x}}\prime )|\chi ({\boldsymbol{x}})} \right\rangle } \right).$$

If such an emission happens at some constant rate Λ, we expect the information about this event to pass observers off at infinity at the same rate. Thus, after some small time Δ*t* has passed, we should have a change in the reduced density matrix of:

13 $$\rho ({\boldsymbol{x}},{\boldsymbol{x}}\prime ,{\mathrm{\Delta }}t) - \rho ({\boldsymbol{x}},{\boldsymbol{x}}\prime ,0) = - \rho ({\boldsymbol{x}},{\boldsymbol{x}}\prime ,0){\mathrm{\Lambda \Delta }}t\left( {1 - \left\langle {\chi ({\boldsymbol{x}}\prime )|\chi ({\boldsymbol{x}})} \right\rangle } \right).$$

Dividing by Δ*t* and taking the limit Δ*t* → 0, we have:

14 $$\frac{{\partial \rho ({\boldsymbol{x}},{\boldsymbol{x}}\prime )}}{{\partial t}} = - {\mathrm{\Lambda }}\left( {1 - \left\langle {\chi ({\boldsymbol{x}}\prime )|\chi ({\boldsymbol{x}})} \right\rangle } \right)\rho ({\boldsymbol{x}},{\boldsymbol{x}}\prime ).$$

Our decoherence rate is therefore given by:

15 $$\tau _{\mathrm{D}}^{ - 1} = {\mathrm{\Lambda }}\left( {1 - \left\langle {\chi ({\boldsymbol{x}}\prime )|\chi ({\boldsymbol{x}})} \right\rangle } \right).$$

For Hawking radiation from a large black hole, we generally expect emission of massless species such as photons and gravitons. (Black holes with radii of order 10^−13^*m* or smaller would also radiate massive particles as the temperature would be comparable to or larger than the rest mass of electrons, but we will not treat this case.) These emissions will be into a distribution of states with differing frequencies (or momenta) and angular momentum for each species. Λ will then be the total emission rates for all momenta and mode types for a given species.

We note that our analysis, like all discussions of decoherence, assumes realistic properties related to the arrow of time. These properties are believed by many to ultimately be of cosmological origin^20^ and exclude, for example, the time reverse of the decoherence process.

### Calculating the overlap

Let us now evaluate the inner product term. Following Hornberger et al.^21^, we can express the density matrix for the radiated particles (assuming isotropic emission) in momentum space as:

16 $$\rho _{{\mathrm{radiation}}} = {\int} {\kern 1pt} {\mathrm{d}}{\boldsymbol{q}}\frac{{p(q)}}{{4\pi q^2}}\left| {\boldsymbol{q}} \right\rangle \left\langle {\boldsymbol{q}} \right|,$$

where *p*(*q*) is the probability of emitting a particle with this magnitude of momentum.

In order to account for the emitter being displaced from the origin, we simply act with translation operators:

17 $$\rho _{{\mathrm{radiation}}} = {\int} {\kern 1pt} {\mathrm{d}}{\boldsymbol{q}}\frac{{p(q)}}{{4\pi q^2}}{\mathrm{e}}^{ - i\widehat {\boldsymbol{q}} \cdot {\boldsymbol{x}}/\hbar }\left| {\boldsymbol{q}} \right\rangle \left\langle {\boldsymbol{q}} \right|{\mathrm{e}}^{i\widehat {\boldsymbol{q}} \cdot {\boldsymbol{x}}\prime /\hbar }.$$

If we define our coordinate system so that ***x*** − ***x***′ = Δ*x*$\widehat {\boldsymbol{z}}$ and then change the integral to spherical coordinates, the trace over this density matrix (the overlap between the states) of emitted particles with different emitter positions is then:

18 $$\left\langle {\chi ({\boldsymbol{x}}\prime )|\chi ({\boldsymbol{x}})} \right\rangle = {\int} {\kern 1pt} {\mathrm{d}}q{\int} {\kern 1pt} {\mathrm{d}}\phi{\int}{\mathrm{d}}\,{\mathrm{cos}}(\theta ){\mathrm{e}}^{ - iq{\mathrm{\Delta }}x{\mathrm{cos}}(\theta )/\hbar }\frac{{p(q)}}{{4\pi }}.$$

Integrating over the angular variables gives us:

19 $$\left\langle {\chi ({\boldsymbol{x}}\prime )|\chi ({\boldsymbol{x}})} \right\rangle = {\int} {\kern 1pt} {\mathrm{d}}q\;{\mathrm{sinc}}\left( {\frac{{{\mathrm{\Delta }}xp}}{\hbar }} \right)p(q).$$

In order to match standard notations, let us change variables from momenta *p* to angular frequency *ω*:

20 $$\left\langle {\chi ({\boldsymbol{x}}\prime )|\chi ({\boldsymbol{x}})} \right\rangle = {\int} {\kern 1pt} {\mathrm{d}}\omega {\mathrm{sinc}}\left( {\frac{{{\mathrm{\Delta }}x\omega }}{c}} \right)p(\omega ).$$

where $p(\omega ) = cp(q)/\hbar$. Again following Hornberger et al.^21^, we define the rate of particle emission per *ω* as:

21 $${\mathrm{\Lambda }}(\omega ) = p(\omega ) \ast {\mathrm{\Lambda }}_{{\mathrm{total}}},$$

with

22 $${\mathrm{\Lambda }}_{{\mathrm{total}}} = {\int} {\kern 1pt} {\mathrm{d}}\omega {\mathrm{\Lambda }}(\omega ).$$

In this notation, we can express our overlap as:

23 $$\left\langle {\chi ({\boldsymbol{x}}\prime )|\chi ({\boldsymbol{x}})} \right\rangle = \frac{1}{{{\mathrm{\Lambda }}_{{\mathrm{total}}}}}{\int} {\kern 1pt} {\mathrm{d}}\omega {\mathrm{\Lambda }}(\omega ){\mathrm{sinc}}\left( {\frac{{{\mathrm{\Delta }}x\omega }}{c}} \right).$$

### Black hole decoherence rate

Specializing to a Schwarzschild black hole, Page^10^ tells us that the emission rate per frequency of the Hawking radiation should be:

24 $${\mathrm{\Lambda }}(\omega ) = \mathop {\sum}\limits_{{\mathrm{s,l,m}}} \frac{1}{{2\pi }}{\mathrm{\Gamma }}_{{\mathrm{s,l,m}}}\left( {{\mathrm{e}}^{4\pi \omega R_{\mathrm{S}}/c} - 1} \right)^{ - 1},$$

where the Γ's are the graybody factors. Using the geometrical optics approximation for a massless species with two polarization degrees of freedom, we have^10^:

25 $$\mathop {\sum}\limits_{{\mathrm{s,l,m}}} {\kern 1pt} {\mathrm{\Gamma }}_{{\mathrm{s,l,m}}} = 2 \ast \frac{{27R_{\mathrm{S}}^2\omega ^2}}{{c^2}}.$$

Our emission rate per frequency interval (summed over the modes) is then:

26 $${\mathrm{\Lambda }}(\omega ) = \frac{{27R_{\mathrm{S}}^2\omega ^2}}{{\pi c^2}}\left( {{\mathrm{e}}^{4\pi \omega R_{\mathrm{S}}/c} - 1} \right)^{ - 1}.$$

This gives a total emission rate of:

27 $${\mathrm{\Lambda }}_{{\mathrm{total}}} = \frac{{27\zeta (3)c}}{{32\pi ^4R_{\mathrm{S}}}}.$$

The corresponding overlap is:

28 $$\left\langle {\chi ({\boldsymbol{x}}\prime )|\chi ({\boldsymbol{x}})} \right\rangle = \frac{{i\pi R_{\mathrm{S}}\left( {\psi ^{(1)}\left( {1 + \frac{{i{\mathrm{\Delta }}x}}{{4\pi R_{\mathrm{S}}}}} \right) - \psi ^{(1)}\left( {1 - \frac{{i{\mathrm{\Delta }}x}}{{4\pi R_{\mathrm{S}}}}} \right)} \right)}}{{{\mathrm{\Delta }}x\zeta (3)}}.$$

This gives the following decoherence rate (Eq. (7)):

29 $$\begin{array}{*{20}{l}} {\tau _{\mathrm{D}}^{ - 1}({\mathrm{\Delta }}x)} \hfill & = \hfill & {\frac{{27c\zeta (3)}}{{32\pi ^4R_{\mathrm{S}}}}} \hfill \\ {} \hfill & {} \hfill & { + \frac{{27ic\left( {\psi ^{(1)}\left( {1 - \frac{{i{\mathrm{\Delta }}x}}{{4\pi R_{\mathrm{S}}}}} \right) - \psi ^{(1)}\left( {1 + \frac{{i{\mathrm{\Delta }}x}}{{4\pi R_{\mathrm{S}}}}} \right)} \right)}}{{32\pi ^3{\mathrm{\Delta }}x}}.} \hfill \end{array}$$

We note that the overlap between two field-theoretic configurations in the 2D “CGHS” (Callen-Giddings-Harvey-Strominger) toy model representing a superposition of a black hole and a white hole or a black hole and a vacuum was computed^22^. The analog of Hawking radiation in that tractable but unphysical model is very different (e.g., its temperature does not depend on black hole mass, and a superposition representing a black hole with two different masses would not decohere). Moreover *x* = *x*′ (i.e., non-local superpositions were not considered in Ref. ^22^). That being said, these CGHS 2D calculations can be argued to be related by analogy to a Schwarzschild case. They also present the decoherence by Hawking radiation as something fundamental and unavoidable, just as we do. However, aside from noting these very broad parallels, we see no concrete way of relating these CGHS 2D calculations to our result.

## Supplementary information

Peer Review

## Data Availability

Data sharing is not applicable to this article as no datasets were generated or analyzed.
