# Supplementary material for: Decoherence of black hole superpositions by Hawking radiation
Source: Nat Commun. 2019 Mar 4;10:1024. doi: 10.1038/s41467-019-08426-4 (PMC6399226; doi:10.1038/s41467-019-08426-4)
Supplement: Supplementary file 1 — Peer Review [file 41467_2019_8426_MOESM1_ESM.pdf]

Reviewers' comments:

Reviewer #1 (Remarks to the Author):

The understanding of the quantum-to-classical transition is a central ingredient for any interpretation of quantum theory. Most experts agree that decoherence is essential in this transition. For ordinary quantum mechanical systems, decoherence is theoretically and experimentally well understood.

The authors of this paper (which is similar to their arXiv paper 1708.09353) apply decoherence to more exotic situations, namely, superpositions of black holes. They show that the Hawking radiation of the black hole serves as an unavoidable decohering environment, because the black hole can never be isolated from this radiation. The authors calculate the decoherence rate for two cases: first, a black hole being immersed in a bath of thermal radiation with Hawking temperature and second, the case of emission of Hawking radiation into vacuum. They find that the decoherence rates are independent of h-bar, in contrast to the rates usually found in quantum mechanics. The main message of this paper is that Hawking radiation by itself is sufficient to decohere black hole superpositions, and that no other environment is needed.

The calculations are straightforward generalizations from quantum mechanical decoherence formulae found in the literature, e.g. the scattering of photons from dielectric spheres. I believe that these calculations and the results are correct. Various approximations are made (e.g. the geometric optics limit), but they are under control and make physical sense.

The results are new and interesting and eventually deserve publication. I have, however, some comments that the authors may wish to address before doing so.

First, although I agree that the decoherence by Hawking radiation plays a fundamental role due to its unavoidability, there are other important environments that might lead to stronger decoherence. One is the ubiquitous cosmic microwave background (CMB) radiation (see e.g. Ref. [11]). It would be interesting to estimate the decoherence rates from the CMB, in particular for the black holes discovered from gravitational radiation (Ref. [18,19]), and to compare them with the rates calculated in this paper. This may also be relevant for the decoherence of the nonlocalities that arise from chaotic trajectories (as mentioned on p.9).

My second point concerns the h-bar independence of the decoherence rates. Its origin lies in the fact that the Hawking temperature is proportional to h-bar, which results from the fact that the Planck spectrum for Hawking radiation is h-bar independent. From this point of view, their result is not suprising - the temperature itself is a genuine quantum effect. The authors may add some clarifying sentences on this.

My third point deals with the remarks after Eq. (29).

It it true that the CGHS model is astrophysically irrelevant, but it may serve as a good analogy for the Schwarzschild case by translating one of the CGHS parameters into the mass. The superpositions considered there are indeed not in real space; the formalism is that of quantum field theory, so the nonlocality is in field space. But the main idea - the unavoidability of decoherence by Hawking radiation - is the same as here. Again, the authors may wish to be more precise about this point.

Finally, two typos: in Eqs (7) and (8), it should read R_S instead of RS; The sentence after Eq. (10) should end with a full stop, not a comma.

We resubmit our paper “Decoherence of Black Hole Superpositions by Hawking Radiation” for your consideration. We thank the referee for careful reading of our paper (that resulted, in addition to more substantial comments, in catching a few typos!).

We agree with, and have followed all of the referee’s recommendations. We have made edits to the manuscript (highlighted) to address each of them. We note that the first two suggestions are handled in two consecutive paragraphs, as the discussion of the decoherence due to the cosmic microwave background provides us with an ideal motivation to note the reasons for the disappearance of the Planck constant from the formula for decoherence rate due to Hawking radiation.

Here are our point by point responses:

> “Reviewers' comments:
>
> Reviewer #1 (Remarks to the Author):
>
> The understanding of the quantum-to-classical transition is
> a central ingredient for any interpretation of quantum theory.
> Most experts agree that decoherence is essential in this transition.
> For ordinary quantum mechanical systems, decoherence is theoretically
> and experimentally well understood.
>
> The authors of this paper (which is similar to their arXiv paper 1708.09353)
> apply decoherence to more exotic
> situations, namely, superpositions of black holes. They show that
> the Hawking radiation of the black hole serves as an unavoidable
> decohering environment, because the black hole can never be isolated
> from this radiation. The authors calculate the decoherence rate for two
> cases: first, a black hole being immersed in a bath of thermal radiation
> with Hawking temperature and second, the case of emission of Hawking radiation

into vacuum. They find that the decoherence rates are
independent of h-bar, in contrast to the rates usually found in
quantum mechanics. The main message of this paper is that Hawking radiation
by itself is sufficient to decohere black hole superpositions, and
that no other environment is needed.

The calculations are straightforward generalizations from quantum
mechanical decoherence formulae found in the literature, e.g. the scattering of
photons from dielectric spheres. I believe that these calculations and
the results are correct. Various approximations are made (e.g. the geometric
optics limit), but they are under control and make physical sense.

The results are new and interesting and
eventually deserve publication. I have, however, some comments that the authors
may wish to address before doing so.

First, although I agree that the decoherence by Hawking radiation plays
a fundamental role due to its unavoidability, there are other
important environments that might lead to stronger decoherence.
One is the ubiquitous
cosmic microwave background (CMB) radiation (see e.g. Ref. [11]).
It would be interesting to estimate the decoherence rates from the CMB,
in particular for the black holes discovered from gravitational
radiation (Ref. [18,19]), and to compare them with the rates
calculated in this paper. This may also be relevant for the
decoherence of the nonlocalities that arise from chaotic
trajectories (as mentioned on p.9)."

We have added some comments and a rough estimate of the scale of the decoherence rate from the CMB. Please see lines 159-167 for the relevant changes.

"My second point concerns the h-bar independence of the decoherence rates.
Its origin lies in the fact that the Hawking temperature is
proportional to h-bar, which results from the fact that
the Planck spectrum for Hawking radiation is h-bar independent.
From this point of view, their result is not suprising - the temperature
itself is a genuine quantum effect. The authors may add some clarifying
sentences on this."

We agree with this sentiment. Please refer to lines 168-170 for the relevant changes.

"My third point deals with the remarks after Eq. (29).
It it true that the CGHS model is astrophysically irrelevant, but
it may serve as a good analogy for the Schwarzschild case by translating
one of the CGHS parameters into the mass. The superpositions considered there
are indeed not in real space; the formalism is that of quantum field theory,

> so the nonlocality is in field space. But the main idea - the unavoidability
> of decoherence by Hawking radiation - is the same as here.
> Again, the authors may wish to be more precise about this point."

We have expanded our discussion of the relationship and relevance of the CGHS paper, specifically acknowledging more of the parallels between the works. The relevant changes are on lines 245-248.

> "Finally, two typos: in Eqs (7) and (8), it should read R_S instead of RS;
> The sentence after Eq. (10) should end with a full stop, not a comma."

Thank you! We have corrected these typos and a few others we have noticed.

The other modification we have made is the inclusion of the required "Data Availability" statement on lines 250-251.

Once again, we appreciate the time and effort that both of you have put into evaluating this paper.

REVIEWERS' COMMENTS:

Reviewer #1 (Remarks to the Author):

The authors have carefully revised their paper in response to my comments. In my opinion, the paper is suitable for publication in its present form.

“Reviewer #1 (Remarks to the Author):

The authors have carefully revised their paper in response to my comments. In my opinion, the paper is suitable for publication in its present form.”

We appreciate this evaluation and naturally agree. Thank you.
